# The Resolution of Severe Iron-Deficiency Anemia After Successful Eradication of *Helicobacter pylori* in Teenagers

**DOI:** 10.1097/PG9.0000000000000238

**Published:** 2022-08-16

**Authors:** Seiichi Kato, Benjamin D. Gold, Ayumu Kato

**Affiliations:** From the * Department of Pediatrics, Tohoku University School of Medicine, Sendai, Japan; † Kato Children’s Clinic, Natori, Japan; ‡ Gi Care for Kids, Children’s Center for Digestive Healthcare, LLC, Atlanta, GA; § Department of General Pediatrics and Gastroenterology, Miyagi Children`s Hospital, Sendai, Japan.

**Keywords:** case series, follow-up study, gastritis, iron deficiency, sports activities

## Abstract

Long-term follow-up studies with *Helicobacter pylori* eradication therapy in children with *H. pylori*-associated iron-deficiency anemia (IDA) are scarce. We investigated whether successful *H. pylori* eradication would result in maintaining resolution of recurrent and/or refractory IDA in a cohort of teenagers in Japan.

**Methods:**

In this case series, 7 *H. pylori*-infected patients with recurrent and/or refractory IDA (12–16 y old) received successful eradication therapy and were then followed for a median of 20 months (range, 9–76 mo) after oral iron supplementation therapy (1–4 mo) was discontinued. Five patients of our study cohort participated in rigorous sports activities.

**Results:**

No visual appearance of ulcerations or erosions was found by esophagogastroduodenoscopy. In all patients studied, the gastric biopsies showed histological evidence of chronic gastritis without significant atrophy and intestinal metaplasia. Compared with the baseline (median values: hemoglobin, 6.3 g/dL; serum iron, 9 μg/dL; serum ferritin, 1.5 ng/mL), values of hemoglobin (*P* < 0.001), serum iron (*P* < 0.005), and ferritin (*P* < 0.001) significantly increased, on average, 2–3 months after eradication therapy and these iron indices were maintained at the same or higher levels at the endpoint of follow-up (median values: 14.2 g/dL, 102 μg/dL, and 29.3 ng/mL, respectively). No patient had recurrence of IDA at the time of final follow-up.

**Conclusions:**

*H. pylori* infection can be closely associated with recurrent or refractory IDA in teenage children. It is speculated that increased iron demands as a result of growth spurt in adolescents may play a synergistic role in combination with *H. pylori* in the pathogenesis of IDA.

**What Is Known**

- Persistent infection with *Helicobacter pylori* has been associated with recurrent or refractory iron deficiency and/or iron-deficiency anemia (IDA) in children.
- A number of mechanisms underlying iron deficiency and/or IDA as caused by *H. pylori* infection have been proposed. However, long-term studies of *H. pylori*-associated IDA in children are lacking, which confirm and validate these postulated etiologic mechanisms.

**What Is New**

- Successful *H. pylori* eradication leads to long-term resolution of recurrent or refractory IDA in teenaged children.
- In such pediatric populations, increased iron demand in adolescent children could play a synergistic role in conjunction with *H. pylori* in the pathogenesis of IDA due to growth spurts and/or sports activities.

## INTRODUCTION

Persistent colonization with *Helicobacter pylori* causes chronic inflammation in the stomach of infected children and adults. *H. pylori*-associated gastric and duodenal inflammation leads to the development of duodenal and to a lesser extent gastric ulcers, gastric adenocarcinoma, and mucosa-associated lymphoid tissue lymphoma in a subset of infected individuals (1). Furthermore, meta-analyses have demonstrated that extragastrointestinal disease, iron-deficiency anemia (IDA), particularly chronic and unexplained (ie, those individuals in whom no obvious cause for the iron deficiency or anemia was identified with investigation), is closely associated with *H. pylori* infection (2,3). In a meta-analysis by Hudak et al (3), *H. pylori* infection significantly increases likelihood of IDA and iron deficiency with a pooled odds ratio of 1.33 and 1.15, respectively. The Maastricht V/Florence Consensus Report recommends eradication therapy for IDA patients with *H. pylori* infection (4). On the other hand, *H. pylori* infection prevalence increases in the pre-teen and adolescent age groups of childhood, and IDA is also more prevalent in those age groups, irrespective of the etiology (5). The most recent pediatric guidelines also recommend *H. pylori* testing and eradication for children in whom IDA is recurrent or refractory to iron supplementation and the other cause have not been identified (5,6).

In *H. pylori*-infected adults, IDA may be caused by impaired iron absorption due to reduced gastric acidity and ascorbic acid concentration related to *H. pylori* atrophic gastritis (7) or increase serum hepcidin (8). However, Japanese children with *H. pylori* infection show no significant gastric atrophy (9) and consequently no reduction of gastric acid secretion (10). Thus, the mechanism(s) for *H. pylori*-associated IDA in childhood remains poorly understood. It is well known that IDA during first 5 years of a child’s life is mainly associated with increased iron demand due to their rapid growth (11). In fact, a randomized control study reported that *H. pylori* is not a cause for treatment failure of iron supplementation in children 2–5 years of age (12). On the other hand, *H. pylori*-associated IDA frequently occurs in school-aged children, suggesting that increased iron demand due to growth spurt and/or participation in sports-related activities is also important in the pathogenesis of IDA (13,14). Of the bacterial factors involved in IDA development, enhanced iron uptake from the host by the microorganisms has been hypothesized (14). Iron is essential for cell growth and maintenance not only in human hosts but also in the primarily metabolic processes of a number of bacteria, in particular *H. pylori*. Recently, sialic acid-binding adhesin gene *sabA* in *H. pylori* has been reported to be as an IDA-associated gene in childhood and adolescence (15). The known genes related to iron uptake/regulation including *fecA*, *fur*, *feoB*, or *pfr* genes were not associated with IDA (15). Of the host factors, it was reported that childhood IDA risk due to *H. pylori* infection may be associated with interleukin-1β gene polymorphism (16).

Thus, although *H. pylori* causality on childhood IDA is thought to be established, long-term follow-up studies, particularly those over 6 months, with eradication therapy and cure of infection confirmation by testing for *H. pylori* infection are scarce and/or nonexistent. The present study reports long-term efficacy with *H. pylori* eradication and concurrent maintenance of IDA in a small cohort of children with previous recurrent and/or refractory IDA.

## METHODS

### Patients

Seven pediatric patients with recurrent and/or refractory IDA were referred to Tohoku University Hospital (Table 1). At consultation, IDA was recurrent in 5 patients, refractory to iron supplementation therapy in one, and recurrent and refractory in one. Patients numbers 3 and 4 were sibling cases. Five patients participated in intensive athletic and sport club activities. In all patients, anthropometrics, including the body height and weight were within normal ranges of corresponding age groups (Table 1). Gastrointestinal symptoms in this cohort included abdominal pain, hematemesis, and black and tarry (ie, melanotic) stools, or other objective signs of gastrointestinal bleeding. In addition, insufficient intake of bioavailable iron in the daily diet were not reported in any of the patients included in this cohort.

**TABLE 1.** Patient summary and hematological data

Case no.	Age(y)/sex	Body height (cm)/weight (kg)	Sports club	Endoscopic diagnosis	Status of IDA*	Hematological data†			
						RBC (×10^4^/µL)	Hb (g/dL)	Iron (µg/dL)	Ferritin (ng/mL)
1	15/F	152/44	Basketball	NG	1st	273	6.2	10	5.6
2	12/M	152/40	None	NG	1st	420	6.5	20	3.4
3	16/M	173/61	Soccer	Gastritis	4th and refractory	408	6.9	12	3.8
4	14/F	139/37	Basketball	Gastritis	1st	410	8.3	16	1.5
5	13/M	149/46	Table tennis	NG	1st	371	6.1	6	1.1
6	15/M	141/35	Table tennis	Gastritis	Refractory	392	7.1	10	2.5
7	15/M	165/53	None	NG	1st	359	5.3	8	0.9

Reference normal ranges in male and female: 450–530 × 10^4^ and 410–510 × 10^4^/µL for RBC count, 12.0–17.0 and 12.0–16.0 g/dL for hemoglobin, 54–200 and 48–154 µg/dL for serum iron, and 39.4–340 and 3.6–114 ng/mL for serum ferritin, respectively. F = female; Hb = hemoglobin; IDA = iron-deficiency anemia; M = male; NG = nodular gastritis; RBC = red blood cell.

*1st or 4th recurrence.

†The data represent those when hemoglobin levels were the lowest from the first onset to consultation to the hospital.

### Laboratory and Endoscopic Examinations

In all patients, red blood cells morphology demonstrated microcytosis and hypochromia with low concentrations of serum iron and ferritin (Table 1). Hematological data in Table 1 demonstrated that hemoglobin levels (median, 6.5 g/dL; range, 5.3–8.3 g/dL) were the lowest from the first onset of IDA to consultation at our hospital. Additional hematological indices including white blood cells including differentiation and peripheral platelet counts were normal in all patients. Metabolic and biochemical tests including serum total protein, albumin, electrolytes, and aminotransferase activities, and urinalysis were all normal. All 7 patients showed negative immunochemical (hemoglobin) stool occult tests.

Before eradication therapy, all 7 patients underwent esophagogastroduodenoscopy (EGD) and gastric biopsies. Duodenal biopsy during diagnostic EGD was not routinely done because Celiac disease is extremely rare in Japan. Using 3.5‰ of a cutoff value (17), ^13^C-urea breath test confirmed positive results in all patients. Endoscopic examination showed nodularity with corresponding histologic gastritis in 4 of the 7 patients. Hemorrhagic lesions such as erosions or ulcerations were not detected between the esophagus and descending part of the duodenum in any patients. Biopsy tests including urease test, histology, and culture were all positive for *H. pylori* infection. Biopsy specimens from gastric body and antrum were histologically evaluated according to the updated Sydney classification system (18).

### Eradication Therapy and Iron Supplementation

All patients were treated with 7-day course of lansoprazole-based triple regimens with amoxicillin plus clarithromycin or metronidazole (6). In addition, the patients received oral iron supplementation therapy with sodium ferrous citrate (Ferromia) (50–100 mg/d as iron) until 1–4 months after completion of eradication therapy.

### Statistical Analyses

For comparison of hematological data among the baseline, 2–3 months after eradication therapy and the final follow-up, paired *t* test was performed and *P* < 0.05 was considered statistically significant.

### Ethical Considerations

In this study, histological examination was approved by the Ethics Committee of Tohoku University School of Medicine (No. 2001-269). The data were anonymous and informed consent was obtained for the patients and/or their parents.

## RESULTS

### Histological Study

EGD showed no ulcerations or erosions in all patients. Nodular gastritis was endoscopically diagnosed in 4 patients, whereas such findings were not detected in other 3 patients (Table 1). All 7 patients showed histological evidence of chronic gastritis. Based on the updated Sydney classification system (18), median grades of inflammation of gastritis in the antrum and corpus were 3 (range, 2–3) and 1 (range, 0–2), respectively, and those of activity of gastritis were 2 (range, 1–2) and 1 (range, 0–2), respectively. Grades of atrophy ranged between 0 and 1 (median, 0) in the antrum and were all 0 in the corpus. No intestinal metaplasia was observed in any patients.

### Hematological Course

**FIGURE 1.**
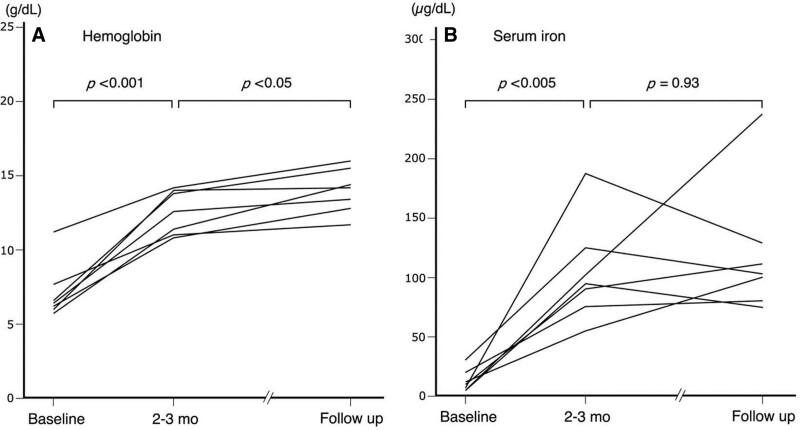
Change of values of hemoglobin and serum iron. Hematological data before eradication therapy (Baseline), 2–3 months after the therapy (2–3 mo), and the endpoint of follow-up (follow up) with a median of 20 months (range, 9–76 mo) after iron supplementation therapy was stopped in 7 patients studied: (A) hemoglobin, (B) serum iron.

^13^C-urea breath test was performed 4 weeks or more after eradication therapy was completed and negative results demonstrated successful eradication of *H. pylori* infection in all patients. The patients were subsequently followed until a median of 20 months (range, 9–76 mo) after iron supplementation therapy was stopped. With regard to hemoglobin, the levels at 2–3 months after eradication therapy (median, 12.5 g/dL; range, 10.8–14.2 g/dL) significantly increased from those at the baseline (median, 6.3 g/dL; range, 5.8–11.2 g/dL) (*P* < 0.001) (Fig. 1). Similarly, there was a significant difference in serum iron between the baseline (median, 9 µg/dL; range, 5–30 µg/dL) and 2–3 months follow-up (median, 95 µg/dL; range, 55–205 µg/dL) (*P* < 0.005). A significant difference was also shown in serum ferritin between the baseline (median, 1.5 ng/mL; range, 0.9–6.8 ng/mL) and 2–3 months follow-up (median, 12.1 ng/mL; range, 8.9–23.9 ng/mL) (*P* < 0.001).

**FIGURE 2.**
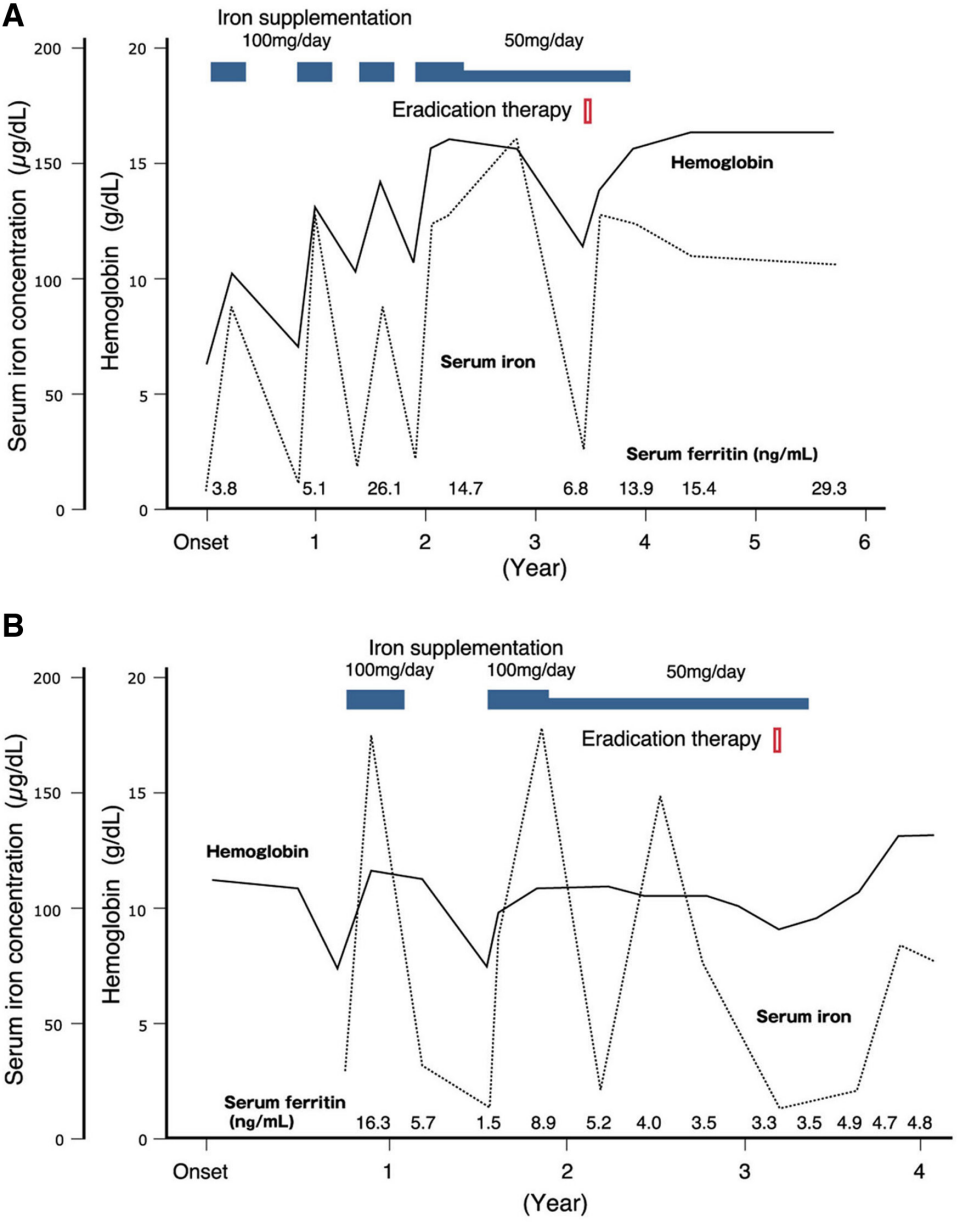
Clinical and hematological course. Clinical course from the first onset of IDA to the endpoint of follow-up: (A) Case number 3, (B) Case number 6. Solid and broken lines represent values of hemoglobin and serum iron, respectively. IDA = iron-deficiency anemia.

Compared with 2–3 months after eradication therapy, levels of hemoglobin significantly increased at the endpoint of follow-up (median, 14.2 g/dL; range, 10.8–16.1 g/dL) (*P* < 0.05) but those of serum iron at the endpoint did not differ (median, 102 µg/dL; range, 76–239 µg/dL) (*P* = 0.93) (Fig. 1). There was no significant in levels of serum ferritin difference between 2 and 3 months and the endpoint (median, 29.3 ng/mL; range, 3.9–102.1 ng/mL) (*P* = 0.15). Clinical and hematological course of case numbers 3 and 6 as a representation of the 7 cases was illustrated in Figure 2A, B.

## DISCUSSION

Our study is the first pediatric case series of *H. pylori*-infected children that clearly demonstrated long-term follow-up documentation of the persistence of normal hemoglobin and hematocrit as well as iron indices after the successful eradication of *H. pylori* infection, especially in a pediatric cohort with recurrent and/or refractory IDA. In addition, the fact that low baseline levels of hemoglobin and serum iron dramatically improved after eradication therapy and were maintained without decrease of these indices at the final follow-up provide evidence for causality between *H. pylori* infection and IDA, and, likely represent the typical course of *H. pylori*-associated IDA. In 1 case series (19), 9 pediatric patients with successful eradication of *H. pylori* were followed between 6 and 24 months. Hematological indices improved 6 weeks after eradication therapy: felt by the authors to be 2 weeks after iron supplementation was stopped. However, unfortunately, follow-up data in this case series were not reported (19). The median follow-up for our cohort was 20 months, which is significantly longer than standard of care for IDA. Our study definitively showed that hematological parameters of IDA such as hemoglobin, serum iron and ferritin both normalize and persist at normal ranges at long-term follow-up periods after *H. pylori* eradication and completion of iron supplementation therapy.

Moreover, our study showed that hemoglobin was observed to be at significantly higher levels at final follow-up than at 2–3 months after eradication therapy, whereas serum iron and ferritin did not differ between these follow-up points. These results indicate that in infected schoolchildren whose *H. pylori* infection was successfully eradicated, normalization of iron metabolism/homeostasis persists for long periods of time, even after iron supplementation therapy was stopped. Taken together, our study observations in our small cohort of children provide compelling evidence for the key role of *H. pylori* infection in IDA of childhood. In a review article (13), it was suggested that *H. pylori* eradication therapy without iron supplementation leads to cure of IDA in children. The present study demonstrated that recurrent or refractory aspects of childhood IDA is closely associated with persistent *H. pylori* infection, thus, our study provides additional data supporting the hypothesis underlying causality of IDA by *H. pylori* infection.

In the present study, none of the patients had visually apparent hemorrhagic mucosal lesions. Hemorrhagic gastritis was a consistent and key finding in the Alaska native population who have a long history of refractory iron deficiency despite diets high in bioavailable iron and in whom *H. pylori* infection is also quite prevalent and thus a high degree of association (20). In children who underwent upper gastrointestinal endoscopy, incidence of anemia was significantly higher in *H. pylori*-positive group than in the negative group, whereas there were no significant differences in incidence of hematemesis and tarry stool between both groups (21). In addition, the majority of *H. pylori*-infected pediatric patients show chronic gastritis including endoscopically evident nodularity without any hemorrhagic lesions (21). Blood loss from the gastrointestinal mucosa does not appear to be the primary pathogenetic cause of *H. pylori*-associated IDA in Alaska native children. Furthermore, all patients studied showed neither significant gastric atrophy nor intestinal metaplasia, compatible with a recently published Japanese multicenter study (9). It is thought that the cohort of patients evaluated in our study do not have significant reduction of gastric acid secretion caused by mucosal atrophy. Thus, this mechanism is likely not involved in the pathogenesis of recurrent or refractory IDA in Japanese teenagers. Considering facts mentioned above, it is concluded that *H. pylori* itself plays a central role in pathogenesis of IDA including recurrent or refractory cases in teenagers in Japan.

In addition, and of note in our small cohort of children, it is important to recognize that 5 patients participated in extracurricular activities, especially elite athletics and intense sports club activities. Choe et al (22) demonstrated that the prevalence of *H. pylori*-associated IDA is higher in adolescent female athletes than in the control group. However, in adolescent female athletes, a significant difference between *H. pylori*-positive and -negative groups and IDA was not found (23). Adolescents are susceptible to iron deficiency because of their high iron demand with sustained increased growth during that age group, showing a trend that females are more susceptible than male due to higher iron demand as a results of menstrual blood loss (22,24). On the other hand, a meta-analysis demonstrated no such association in adult populations (2). Review articles showed that *H. pylori*-associated IDA frequently occurs in children and adolescents, especially who have sports club activities (13,14).

Limitations of the present study include the small number of the patients and absence of the control group. In addition, the present study has no data on the menstrual bleeding in 2 female patients, which is an important casual factor of IDA. Therefore, it is difficult to reach a conclusion that intensive sports activities, play some role in the development of *H. pylori*-associated IDA. And furthermore, in our study, duodenal biopsy was not routinely obtained because Celiac disease is extremely rare in Japan. Thus, any type of duodenal pathology that might contribute to the iron deficiency, for example, Celiac disease or Crohn’s disease would have been missed. However, if either of these etiologies had been present and had contributed to the IDA, then we would assume that since the treatment was not initiated for either of these etiologies and the IDA resolved with *H. pylori* eradication, it is less likely that Celiac disease or Crohn’s disease was missed. We believe that in our unique cohort of Japanese children increased daily iron demand due to growth spurt and/or sports activities plays a synergistic role for a bacterial factor of *H. pylori* infection in development of childhood and adolescent IDA.

## ACKNOWLEDGMENTS

We thank Mr Katsuhisa Hashimoto for assistance preparing the figures.
